# Development of Gene Expression Markers of Acute Heat-Light Stress in Reef-Building Corals of the Genus *Porites*

**DOI:** 10.1371/journal.pone.0026914

**Published:** 2011-10-26

**Authors:** Carly D. Kenkel, Galina Aglyamova, Ada Alamaru, Ranjeet Bhagooli, Roxana Capper, Ross Cunning, Amanda deVillers, Joshua A. Haslun, Laetitia Hédouin, Shashank Keshavmurthy, Kristin A. Kuehl, Huda Mahmoud, Elizabeth S. McGinty, Phanor H. Montoya-Maya, Caroline V. Palmer, Raffaella Pantile, Juan A. Sánchez, Tom Schils, Rachel N. Silverstein, Logan B. Squiers, Pei-Ciao Tang, Tamar L. Goulet, Mikhail V. Matz

**Affiliations:** 1 The University of Texas at Austin, Austin, Texas, United States of America; 2 Department of Zoology, Tel Aviv University, Tel Aviv, Israel; 3 The University of Mauritius and The Biodiversity and Environment Institute, Reduit, Mauritius; 4 Rosenstiel School of Marine and Atmospheric Science (RSMAS), University of Miami, Miami, Florida, United States of America; 5 University of Guam Marine Laboratory, UOG Station, Mangilao, Guam; 6 Texas A&M University Corpus Christi, Corpus Christi, Texas, United States of America; 7 Universite de Perpignan, Perpignan, France; 8 Biodiversity Research Center Academia Sinica, Taipei, Taiwan; 9 Department of Biological Sciences, Florida International University, Miami, Florida, United States of America; 10 Department of Biological Sciences, Kuwait University, Safat, Kuwait; 11 Department of Biology, The University of Texas at Arlington, Arlington, Texas, United States of America; 12 Oceanographic Research Institute, Marine Parade, Durban, South Africa; 13 ARC Centre of Excellence and James Cook University, Townsville, Queensland, Australia; 14 Newcastle University, Newcastle upon Tyne, United Kingdom; 15 Australian Institute of Marine Science, Townsville, Queensland, Australia; 16 Departamento de Ciencias Biológicas, Universidad de los Andes, Bogotá, Colombia; 17 Department of Biology, University of Louisiana at Lafayette, Lafayette, Louisiana, United States of America; 18 Department of Biology, University of Mississippi, University, Mississippi, United States of America; King Abdullah University of Science and Technology, Saudi Arabia

## Abstract

Coral reefs are declining worldwide due to increased incidence of climate-induced coral bleaching, which will have widespread biodiversity and economic impacts. A simple method to measure the sub-bleaching level of heat-light stress experienced by corals would greatly inform reef management practices by making it possible to assess the distribution of bleaching risks among individual reef sites. Gene expression analysis based on quantitative PCR (qPCR) can be used as a diagnostic tool to determine coral condition *in situ*. We evaluated the expression of 13 candidate genes during heat-light stress in a common Caribbean coral *Porites astreoides*, and observed strong and consistent changes in gene expression in two independent experiments. Furthermore, we found that the apparent return to baseline expression levels during a recovery phase was rapid, despite visible signs of colony bleaching. We show that the response to acute heat-light stress in *P. astreoides* can be monitored by measuring the difference in expression of only two genes: Hsp16 and actin. We demonstrate that this assay discriminates between corals sampled from two field sites experiencing different temperatures. We also show that the assay is applicable to an Indo-Pacific congener, *P. lobata*, and therefore could potentially be used to diagnose acute heat-light stress on coral reefs worldwide.

## Introduction

Coral reefs are declining globally, detrimentally affecting biodiversity and local economies [1], [2], [3]. Increasingly severe and frequent episodes of elevated seawater temperature, acting synergistically with intense solar irradiation, have led to recurrent devastating coral bleaching events [3], [4]. Coral bleaching is the breakdown of the partnership between the cnidarian host and its symbiotic algae (*Symbiodinium* spp.) [5]. While recovery is possible, the likelihood of coral mortality increases with the duration of stressful conditions [6]. Due to the increasing severity of bleaching events, associated alterations in coral physiology as well as mechanisms to mitigate their effects need to be understood. Physiological responses to bleaching are generally examined under controlled laboratory conditions and responses of natural populations are rarely evaluated *in situ*. For example, prediction of bleaching events is currently based on physical environmental parameters rather than coral condition in response to those parameters. One model employs the combination of the temperature anomaly (HotSpot) and exposure time (Degree Heating Weeks), typically over a 12-week period (http://coralreefwatch-satops.noaa.gov/SBA.html). A method to rapidly query coral stress responses *in situ* would make it possible to directly evaluate how stressful the local environment is, from the coral's point of view. This would help ground-truth the satellite-based systems and further refine our ability to evaluate the risk distribution across environmental gradients as well as among individual reefs sites. Such information would elucidate some of the key aspects of coral ecology, and would aid in prioritizing conservation efforts.

Recently, gene expression analysis has emerged as a powerful tool to study the molecular mechanisms of thermal stress response in corals. The pioneering works of Snell and co-workers identified 32 stress-regulated genes, related to protein synthesis, apoptosis, cell signaling, metabolism, cellular defense and inflammation [7], [8], [9]. This gene panel has been tested both in the lab and the field, to detect expression changes between populations [10] and in a single population through time [11]. More recently, studies using larger-scale microarrays reported genes regulated during bleaching in adult corals [12], [13] and during heat stress in coral larvae [14], [15], revealing additional transcriptional consequences of coral stress including cytoskeleton reorganization, change in Ca^2+^ homeostasis, heat shock protein expression, transposon activity, and down-regulation of immunity components. In addition to these ubiquitous processes, two apparently coral-specific stress responses have been discovered: down-regulation of GFP-like fluorescent proteins [12], [16], [17], [18], and up-regulation of coral-specific small cysteine-rich proteins (SCRiPs) [19].

The previous studies identified stress-induced markers that could be diagnostic of coral stress in the field; however, they have not provided the tools necessary to rapidly assess those markers from multiple field-collected samples. While microarrays are useful for capturing the full range of expression changes induced by a particular environmental stressor, they are not feasible as tools for field applications. A practical diagnostic assay should be based on a minimal number of genes and the least complicated laboratory procedures, as long as they ensure powerful and accurate stress detection. Quantitative PCR (qPCR) can fill the gap between our knowledge of system-wide gene expression patterns in response to stress and the ability to assess the response of multiple individuals rapidly and cost-effectively. Since qPCR assays do not rely on variable and difficult to obtain detection reagents such as polyclonal antibodies, they are more tractable than previously described protein-based techniques [20], [21], resulting in easy replication across laboratories and therefore facilitating their broad practical application. While any molecular assay for stress detection ultimately requires validation through correlated physiological changes and extensive evaluation in the field, it is our opinion that the current challenge for implementing expression-based methods lies in identifying the genes demonstrating the most pronounced and consistent stress response, preferably with a large dynamic range to enable quantification.

A few recent studies question the potential for making meaningful comparisons within and between individual corals based on expression data. Bay *et al*. [18] found few expression differences between populations from different source environments. The authors conclude this was most likely due not to the absence of differences, but to high variance in expression levels among coral colonies within each source population [18]. Seneca *et al*. [22] also observed high inter-individual variation in *A. millepora* in the field, although significant bleaching-related responses in some genes have been detected. Moreover, variability of stress-related gene expression was observed not only between individuals, but also between clonal fragments obtained from the same individual in laboratory experiments with *A. millepora* [23]. Based on the work of [22], [23]. Souter *et al*. [24] designed a multilocus expression assay for thermal stress in *A. millepora*. The results were similar: due to high variation, only two genes exhibited significantly different expression. In a Caribbean congener, however, significant expression differences under thermal stress were detected for 11% of the candidate genes queried [13]. In an evaluation of thermal stress in *Montastraea faveolata*, another Caribbean species, DeSalvo *et al*. [12] report significant expression differences for 21% of their candidate genes during their sampling time course. However, in a second time course experiment published later using the same species and microarray, only 4–6% of genes were differentially expressed [25]. While these studies have detected significant expression changes under thermal stress, the results are largely inconsistent, both within and between species. This suggests that robust stress detection based on gene expression may not be achievable, at least in these systems.

The main goal of this study was to further explore the potential of a number of genes highlighted by previous works to serve as components of a qPCR-based stress detection and quantification assay. Rather than focusing on a single coral species, we sought to develop an assay that would be broadly applicable to an ecologically important group of corals worldwide, and thus could become a universal indicator of reef stress. We chose to focus on the genus *Porites*, as it is the second most speciose coral genus (after *Acropora*), contributing greatly to reef structure all over the world [26]. Importantly, in contrast to *Acropora*, commonly found species of the genus *Porites* are not considered critically endangered, therefore their sampling as bioindicators would be possible. While *Porites* spp. are not as susceptible as other genera to heat-light stress-induced mortality [27], [28], we show that their gene expression patterns are responsive to stress, rendering them a consistent and reliable indicator.

## Methods

### Ethics Statement

Fieldwork in the USA was carried out under permits FKNMS-2009-078 (Experiment 1 and 3), FKNMS-2010-093 (Experiment 2) issued by the Florida Keys National Marine Sanctuary. Fieldwork in Australia was carried out under permit G28854.1 (Experiment 4) issued by the Great Barrier Reef Marine Park Authority and samples were exported to the USA under CITES permit No. 2008-AU-537170.

### Stress Experiments

#### Experiment 1: Heat-Light Stress Expression Patterns

In July 2009, four whole colonies of *Porites astreoides* were obtained from the Florida Keys National Marine Sanctuary nursery in Key West at 10:00 (depth: 2.7 m), and one was also collected from a seawall at the east end of old Bahia Honda Bridge at 15:00 (1 m, 24.655° N, 81.298° W). Water temperatures at the time of sampling were not recorded. Colonies were immediately transported to Mote Tropical Research Lab and allowed to acclimate in a shaded flow-through system supplied with sand-filtered seawater for two days (mean temperature: 27.8±0.7°C). The flow-through system was supplied with additional circulation provided by two submerged pumps. Following the two-day acclimation, colonies were halved. One half was returned to the shaded (control) system, while the other half was placed in a full sun-exposed (treatment) system. Colonies were sampled for gene expression analysis on the fourth day at midday (14:00). StowAway TidbiT temperature data loggers (Onset Computer Corp., Bourne, MA) recorded ambient temperature every two minutes for the duration of the experiment (Fig. S1). Reefs in the Florida Keys annually experience summer maxima of 32°C, and occasionally nearshore sites can reach temperatures in excess of 33°C, which results in annual bleaching. Treatment conditions were deliberately chosen to exceed this natural intensity (two days under large daily temperature variation, reaching 35–36°C at solar maximum, dropping to 28°C at night), under the assumption that any genes failing to show expression change under such extreme conditions are probably poor candidates for stress diagnostics *in situ*. Light intensities were recorded above-water at the time of sampling using the photosynthetic photon flux quantum meter (Spectrum Technologies, Inc., Plainfield, IL), and were found to be 19 µmol-m^−2^-s^−1^ in the control table and 1960 µmol-m^−2^-s^−1^ in the treatment system.

#### Experiment 2: Stress-Recovery Expression Patterns

This experiment was intended to clarify whether the genes regulated in the first experiment responded to the acute stress condition, or reflected cumulative stress over two days of exposure. In August 2010, eight whole colonies of *P. astreoides* at a depth of 1.8 to 3.3 m were collected at 14:00 from an offshore patch reef (24° 31.303′ N, 81° 34.605′ W). Water temperature at the time of sampling was 30.1°C. Colonies were immediately transported to Mote Tropical Research Lab and placed in a shaded flow-through system supplied with sand-filtered seawater (mean temperature: 28.0±0.7°C). On the same day, colonies were quartered using a hammer and chisel, after which all fragments were returned to the shaded flow-through system and allowed to acclimate for four days. The photochemical efficiency of the symbionts was quantified using a pulse amplitude modulated fluorometer (PAM). PAM measurements revealed that the quantum yield of PSII / Φ_PSII_ of *in hospite Symbiodinium* of *P. astreoide*s may have been slightly diminished at the initial sampling/quartering point, but recovered overnight and remained stable throughout the acclimation period (Fig S2). At 09:00 on the fourth day of acclimation, two fragments from each colony were moved to a fully sun-exposed flow-through system. At midday (14:30, “stress” time-point) samples were taken from two fragments per colony, one from the control and one from the treatment system, and immediately processed for gene expression analysis (see *Sampling Procedures*). At 21:00, when the water temperatures of the two flow-through systems became equivalent, PAM measurements were taken of the remaining un-sampled fragments and the un-sampled fragment from the sun-exposed system was returned to the shaded system. On the following day at 14:45 (“recovery” time-point), the remaining two fragments (one heat-light stressed 24 hours earlier and one control) from each colony were sampled and immediately processed for gene expression analysis. Prior to sampling, levels of bleaching were assessed using a color card [29]. The temperature and light in the shaded and exposed systems at midday were similar to those in stress experiment (1), with the temperature difference between treatments reaching 7–8°C and light levels of approximately 20 and 2000 µmol-m^−2^-s^−1^ for control and stress treatments, respectively as measured with the photosynthetic photon flux quantum meter (Spectrum Technologies, Inc., Plainfield, IL) (Fig. S3).

#### Experiment 3: Field validation of double-gene assay

Since stress experiments (1) and (2) were lab-based manipulations, we wanted to test the applicability of the double-gene assay (see *Results*) on field-collected individuals. In July 2009, tissue samples of *P. astreoides* from both an inshore (24°36.296 N, 81°22.745 W; depth: 3.5m; N = 9) and an offshore (24°33.196 N, 81°22.747 W; depth: 4.5m; N = 7) reef were collected (see *Sampling Procedures*). Temperatures at both field sites were measured using StowAway TidbiT temperature loggers (Onset Computer Corp., Bourne, MA) with recordings taken every two minutes. Light measurements are unavailable for this experiment, although in a visual assessment at the time of sampling, the inshore site was more turbid than the offshore site.

#### Experiment 4: Transferability of double-gene assay between species

In order to evaluate the among-species applicability of the actin-Hsp16 double-gene assay (see *Results*), we used material initially collected in December 2008 from earlier experiments with *Porites lobata* from the Great Barrier Reef. Large fragments of five colonies of *P. lobata* were collected from Pioneer bay, Orpheus Island, Australia (18°35.693′S, 146°29.247′E). They were acclimated in indoor tanks supplied with flow-through filtered seawater (28°C) for four days, with metal halide lamps providing the light (∼200–250 µmol-m^−2^-s^−1^) with 12h light/dark cycle. On the fifth day, each large fragment was further divided and three small fragments were placed into each of three treatment tanks (supplied with water at 31°C), while three other small fragments were placed into three control tanks (28°C). The lighting in both the elevated and control temperature tanks was the same as during the acclimation period. After nine days of this treatment, the fragments were sampled for gene expression analysis. Some fragments failed to yield qPCR measurements for a variety of non-biological reasons, resulting in 1–3 replicates per colony per treatment for four colonies, with the fifth colony represented by two control replicates.

### Candidate and Internal Control Gene Selection

Candidate genes were selected for analysis based on differential expression in response to heat stress, copper poisoning, and/or mechanical injury in *P. lobata* and *P. compressa* (Matz, unpublished; the data are available at http://www.bio.utexas.edu/research/matz_lab/matzlab/Data.html). The selected genes also reflect several biological processes shown to be involved in stress response across scleractinians [12], [13], [14], [15]. Nine of our candidate gene primer pairs were originally designed using *P. lobata* and *P. compressa* sequence data. The remaining primer pairs were designed using *P. astreoides* sequence data obtained from the SymBioSys database (http://sequoia.ucmerced.edu/SymBioSys/index.php) (Table 1).

**Table 1:** List of candidate genes used in expression analyses.

Gene Name (Abbreviation)	Biological Process	Forward PrimerReverse Primer	Sequence Information
18s rRNA	Metabolism	F: 5′-AATGATCTATCCCCAGCACG-3′R: 5′-TCCAACCAAAGTCAGGAAGG-3′	*P. lobata*
Alpha B Crystallin(HSP 16)	Heat-shock	F: 5′-TCACAGGAAAACACAGAGCG-3′ R: 5′-GGGTCACGTGCCACTTCTAT-3′	*P. lobata*
Actin	Cytoskeleton	F: 5′-CAGTGTTTCCCTCCATCGTT-3′ R: 5′-CAGTTGGTTACAATGCCGTG-3′	*P. lobata*
Adenosine Kinase (ADK)	Metabolism	F: 5′-AAAGAACCCACTGGAACGTG-3′ R: 5′-CAAATGCCCAGTTTTCTGGT-3′	*P. lobata*
Complement Component C3 (C3)	Immunity	F: 5′-TGTGGCACTACAGGCTCTTG-3′ R: 5′-GACATCAATCGCTCTGCGTA-3′	*P. lobata*
C-type Lectin (Clect)	Immunity	F: 5′-CCCGGTGATACTGTGTCAGA-3′F: 5′-AAATGCCAACCCAAGTAACG-3′	*P. astreoides*
Eukaryotic Initiation Factor 3, Subunit H (EIF3H)	Control gene	F: 5′-TTGATTGATACCAGCCCACA-3′R: 5′-ACAAACTGCTTTGCTTTCCC-3′	*P. astreoides*
Glyceraldehyde-3-Phosphate Dehydrogenase (G3PDH)	Metabolism	F: 5′-TCCATGGACTTCGTTCACAA-3′R: 5′-CAGAAGATCCACCACCCTGT-3′	*P. astreoides*
GFP-like Chromoprotein (Chrom)	Unknown	F: 5′-AGGTGCCACCGTATCACTTC-3′ R: 5′-CACTATTGCCTTTTCGCCAT-3′	*P. lobata*
GTP Binding Protein (GSP2)	Control gene	F: 5′-GACCAGGAAAGAACGTCCAA-3′R: 5′-GGAAAACCGCCATACTCAAA-3′	*P. astreoides*
HSP 60	Heat-shock	F: 5′-CCAGCAGCGGTTTTCTCTTA-3′R: 5′-CGGCAACAGCATCAGTTAAA-3′	*P. astreoides*
HSP 90	Heat-shock	F: 5′-GTTGGGTCGGTCAAACTCTC-3′R: 5′-GAGCATCCGAAGAGTTGGAG-3′	*P. astreoides*
NADH-Dehydrogenase (ND5)	Control gene	F: 5′-AGCATGAATAACAGACCCCG-3′ R: 5′-TTGGGGTGGTTCAAAATGAT-3′	*P. lobata*
60s Ribosomal Protein L11 (Rpl11)	Control gene	F: 5′-TTTCAAGCCCTTCTCCAAGA-3′R: 5′-GACCCGTGCTGCTAAAGTTC-3′	*P. astreoides*
Spondin 2 (Spon2)	Immunity	F: 5′-CACGAGCACAAAAATCATGG-3′R: 5′-GCAGGTCCATTGTCACCTTT-3′	*P. astreoides*
Trans-golgi Network Protein (Tgoln)	Vesicular Protein Transport	F: 5′-GCTGCCTTTTTCTTGACTGC-3′ R: 5′-TCCTGTAGCCTCGCCTTCTA-3′	*P. lobata*
Ubiquitin-like protein 3 (Ubl3)	Protein Degradation	F: 5′-ATGGACTTTTGACCCTCACG-3′R: 5′-ATGGTCGGTTTCTACATGGC-3′	*P. lobata*

Sequence information column indicates database where information was obtained. *P. lobata* information can be found at http://www.bio.utexas.edu/research/matz_lab/matzlab/Data.html. *P. astreoides* information can be found at http://sequoia.ucmerced.edu/SymBioSys/index.php.

Five putative internal control genes were derived from a series RNA-seq experiments conducted on larval families of *Acropora millepora* [27], as being the most stable during long and short--term heat stresses, settlement induction, and metamorphosis. This selection included typical control genes reported for other models: ribosomal protein L11 (RPL11), elongation initiation factor 3H (EIF3H), NADH-dehydrogenase subunit 5 (ND5), glucose-3-phosphate-dehydrogenase (G3PDH), and GTP-binding protein responsible for nuclear organization maintenance (GSP2).

### Primer Design and Validation

Primers were designed using Primer3 (http://primer3.sourceforge.net/). The specificity of each primer pair was verified by gel electrophoresis and melting curve analysis of the amplification product obtained with *P. astreoides* cDNA as a template. Primer efficiencies were determined by amplifying a series of 2-fold dilutions of *P. astreoides* cDNA covering two orders of magnitude of template amount (5 ng to 0.078 ng RNA-equivalent per PCR reaction). The results were plotted as C_P_ vs. log_2_[cDNA], and the primer-specific amplification efficiency *E* (the amplification factor per PCR cycle) was derived from the slope of the regression using formula *E* = 2^−(1/slope)^ [30]. Primer pairs with *E* outside 1.85–2.15 range were redesigned and re-validated. In order to verify primer specificity for coral cDNA, all primer pairs were tested on cDNA from cultured *Symbiodinium* strain B184. No amplification was observed except in the positive control (*Symbiodinium*-specific Hsp70 primers, [31]). The full gene list and primer sequences are given in Table 1.

### Sampling procedures

*Porites* spp. fragments were dabbed with a kimwipe to remove excess seawater. Samples were then taken by scraping off approximately 1 cm^2^ of tissue from the colony surface with a razor blade. In Experiment 1 and 3, samples were taken and immediately placed into a microcentrifuge tube containing 2 ml of 96% ethanol on ice and stored at −20°C. In Experiment 2, the fragments were carried to the lab in a bucket of their respective system water, sampled as described above and immediately processed for RNA isolation. In Experiment 4, the sampled tissue was put into >5 volumes of RNAlater (Ambion) and kept at 0 to −20°C until RNA isolation. A test of all the employed methods of preservation demonstrated that the resulting RNA quality was equivalent to that obtained from freshly extracted material or material snap-frozen in liquid nitrogen. RNA integrity number (RIN) measured on a Bioanalyzer (Agilent) was 8 or higher for all samples, regardless of preservation treatment (Fig. S4).

### RNA isolation and cDNA synthesis

Total RNA was extracted using RNAqueous 4-PCR kit (Ambion). Samples stored in preservative were removed from their tubes and residual liquid was dabbed off using a kimwipe. Each sample was placed in a 25 mm petri dish containing 350 µl Lysis Buffer from the kit and homogenized using a razor blade. Slurries were then transferred to sterile 1.5 ml tubes, an additional 350 µl Lysis Buffer was added and back-pipetted to completely disperse tissue. Samples were then spun for 5 minutes at 5000 rpm in a table centrifuge to precipitate skeleton fragments and other insoluble debris. 700 µl of supernatant was used for extraction following the manufacturers' instructions, with one modification: in the final elution step, the same 25 µl of elution buffer was passed twice through the spin column to maximize the concentration of eluted RNA.

RNA quality was assessed through gel electrophoresis and evaluated based on the presence of the ribosomal RNA bands. If the rRNA bands were poorly visible, the samples were discarded and not included in the analysis (Fig. S5). Total RNA was DNAse treated according to the RNAqueous 4-PCR kit protocol, after which the concentration of RNA was estimated using the Nanodrop 2000 (Thermo-Fisher). For each sample, first strand cDNA was prepared from the amount of total RNA equivalent to 50 ng, using the SmartScribe Reverse Transcriptase kit (Clontech-Takara, Mountain View, CA) and an oligo-dT-containing primer (5′-CGCAGTCGGTACTTTTTTCTTTTTTV-3′). The reaction was then diluted to contain an equivalent of 1 ng/µl of RNA. An aliquot of first strand cDNA equivalent to 1 ng RNA (i.e. 1 µl) was used for each qPCR reaction.

### qPCR and normalization

qPCR reactions were performed in 15 µl volumes using 2x SYBRgreen Master Mix (Roche) in the LightCycler 480 (Roche). Preliminary analyses (CP calling and melting curve analysis) were performed using the GeneScan software (Roche) supplied with the instrument. Each cDNA sample was assayed in duplicate, in independent qPCR runs. The CP values were then converted to values proportional to absolute amounts following [32], using the gene-specific amplification efficiencies (*E*) as the base of the exponent. These values were then log2-transformed to yield Ca values, suitable for linear model analysis (Fig S6). The cumulative formula for these transformations is Ca  =  - CP * log_2_(*E*) (see Supplementary file for derivation). The gene-specific amplification efficiencies (*E*) were derived from analysis of serial dilutions, as explained in the Primer Design and Validation section above. The arithmetic mean of the control genes' Ca values was subtracted from the Ca values of other genes, to yield normalized Ca values. This procedure is mathematically identical to the one suggested by Vandesompele *et al*. [33] in application to the log-transformed absolute amounts, represented by the Ca values. The normalized Ca values were then used for statistical analysis.

### Statistical Analysis

All analyses were carried out using R software [34]. The effects of heat-light stress on candidate gene expression were investigated with a series of linear mixed models using the *lme4* package [35]. For Experiments 1 and 2, each gene was analyzed individually using the normalized Ca value as the response variable. For Experiments 3 and 4, the response variable (D) was the difference between Ca values of actin and Hsp16 (D  =  Ca^Hsp16^ – Ca^Actin^).

For Experiment 1, the effect of heat-light stress was modeled with treatment (control or heat-light stress) as a fixed factor and individual colony as a random factor. For the analysis of Experiment 4, which included replicate samples per colony in replicate tanks, tank effect was included as an additional random factor. For Experiment 2, the effect of heat-light stress was modeled with treatment history (control or treatment) and sampling point (stress or recovery) as fixed factors and individual colony as a random factor.

The nominal P-values for the significance of fixed factors were derived via Markov Chain Monte Carlo (MCMC) simulations using the functions *mcmcsamp* and *HPDinterval* of the *lme4* package. The false discovery rate [36] was controlled at the 5% level using function *p.adjust* in R.

Potential heat-light stress diagnostic genes were identified using principal components analysis (PCA) on only those genes that were common to the “stress” and “stress-recovery” experiments (actin, ADK, C3, Chrom, Hsp16 and Ubl3). PCA was performed using *labdsv* package [37].

## Results

### Verification of internal control genes

To verify the stability of the five putative control genes in the experiments described here, these genes were quantified by qPCR in the actual “stress” (experiment 1) and “stress-recovery” (experiment 2) samples of *P. astreoides,* and ranked by stability using geNorm [33]. The average gene stability values (*M*) of the three most stable genes (RPL11, EIF3H and ND5, Table 1) were 1.22, 1.22, and 1.42, respectively; somewhat higher than typical [32]. To see if this would present a problem, we normalized the control gene data using all five genes as controls and looked at the residual variation. This analysis suggested that the three most stable genes were regulated across experimental conditions by 1.19–1.29 fold, while fluctuating within a given condition by 1.82–1.99 fold. While this variation is clearly non-zero (i.e., the control genes were not perfectly stable), it is notably less than the target gene expression responses that we report here (see below). We therefore deemed the selected three internal control genes suitable for our particular study, given the magnitude of gene expression regulation that we claim to have detected.

### Heat-light stress expression patterns (Experiment 1)

**Figure 1:**
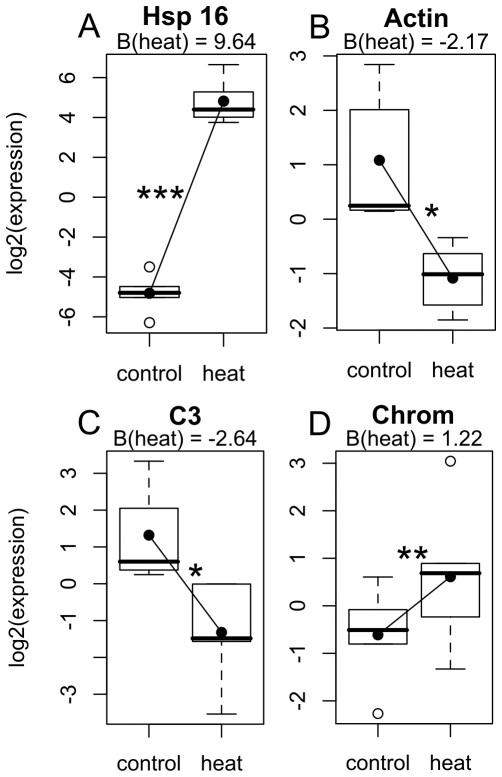
Significant gene expression differences by treatment for Experiment 1 (*Porites astreoides)*. Box-plots show distribution of normalized expression values for all individuals (n = 5 pairs) by treatment. A thick black line indicates the median of normalized expression values. The box represents the inter-quartile range (IQR) between the upper and lower quartile. The whiskers maximally extend 1.5 times beyond the IQR. Open circles indicate outliers. The black circles within each box are predicted values for the condition based on the linear-mixed model results. Lines connecting dots represent the effect of heat-light treatment, given as B at the top of each figure. Effect significances, after applying a multiple-test correction, are represented by (*) = P<0.05, (**) = P<0.01, (***) = P<0.001. Gene abbreviations: C3 =  complement component C3, Chrom  =  GFP-like chromoprotein.

Of the eight genes tested in this experiment, four demonstrated significant expression changes: Hsp16 was dramatically (∼800-fold) up-regulated (P<0.001, Fig 1A). In addition, a GFP-like chromoprotein demonstrated up-regulation under stress (∼2-fold, P<0.01, Fig 1D), while both actin and complement component C3 were down-regulated by ∼4-fold and ∼6-fold, respectively (P<0.01, Fig 1B,C). The heat-light stressed coral fragments did not show observable signs of bleaching after this treatment compared to controls, although the photosynthetic parameters were not monitored in this experiment.

### Stress-recovery expression patterns (Experiment 2)

**Figure 2:**
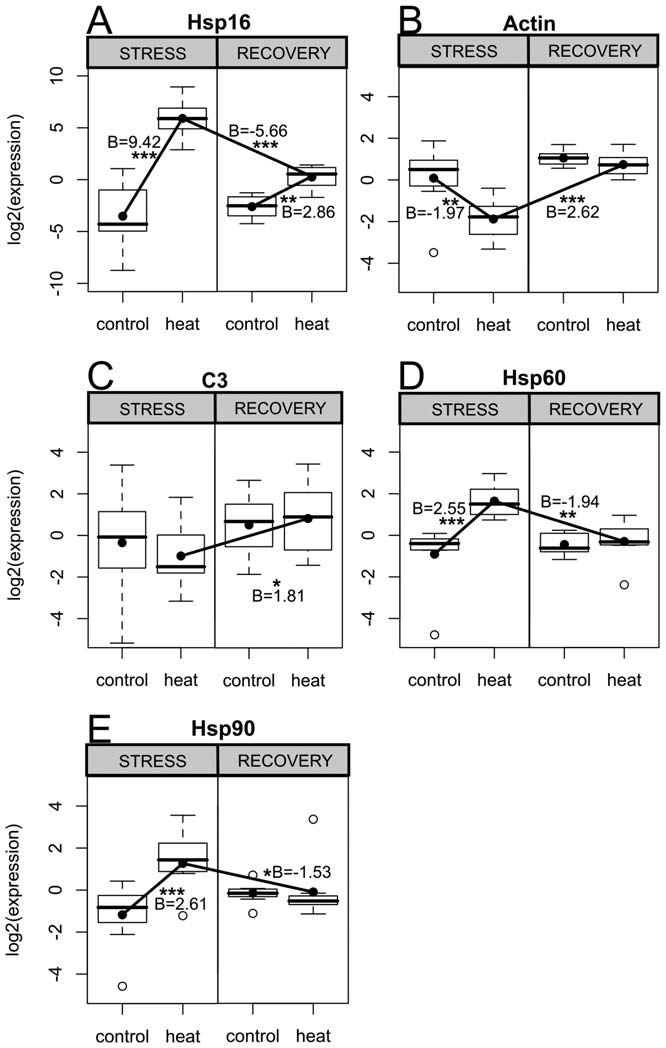
Significant gene expression differences by treatment and/or time in Experiment 2 (*Porites astreoides*). Box-plots show distribution of normalized expression values for all samples (n = 12 pairs). A thick black line indicates the median of normalized expression values. The box represents the inter-quartile range (IQR) between the upper and lower quartile. The whiskers maximally extend 1.5 times beyond the IQR. Open circles indicate outliers. The black circles within each box are predicted values for the condition based on the linear-mixed model results. Lines connecting dots represent significant effects of either time (between stress and recovery) or treatment (between heat and control), given as B next to each line. Effect significances, determined by MCMC simulations, are represented by (*) = P<0.05, (**) = P<0.01, (***) = P<0.001. Gene abbreviations: C3 =  complement component C3.

The expression patterns of both Hsp16 and actin at the “stress” time point recapitulated the results of Experiment 1: Hsp16 was strongly up-regulated (∼700-fold, P_MCMC_<0.001, Fig 2A “stress”), while actin was down-regulated (∼4-fold, P_MCMC_<0.01, Fig 2B “stress”). Complement component C3 also showed a slight trend towards down-regulation (Fig 2C “stress”). The GFP-like chromoprotein, in contrast to Experiment 1, showed no apparent trend. In addition, genes for two large heat-shock proteins (Hsp60 and Hsp90) that we included into our gene panel for the stress-recovery experiment were also up-regulated during the “stress” time point, Hsp60 by ∼4-fold, and Hsp90 by ∼6-fold (P_MCMC_<0.001, Fig. 2D,E “stress”).

**Figure 3:**
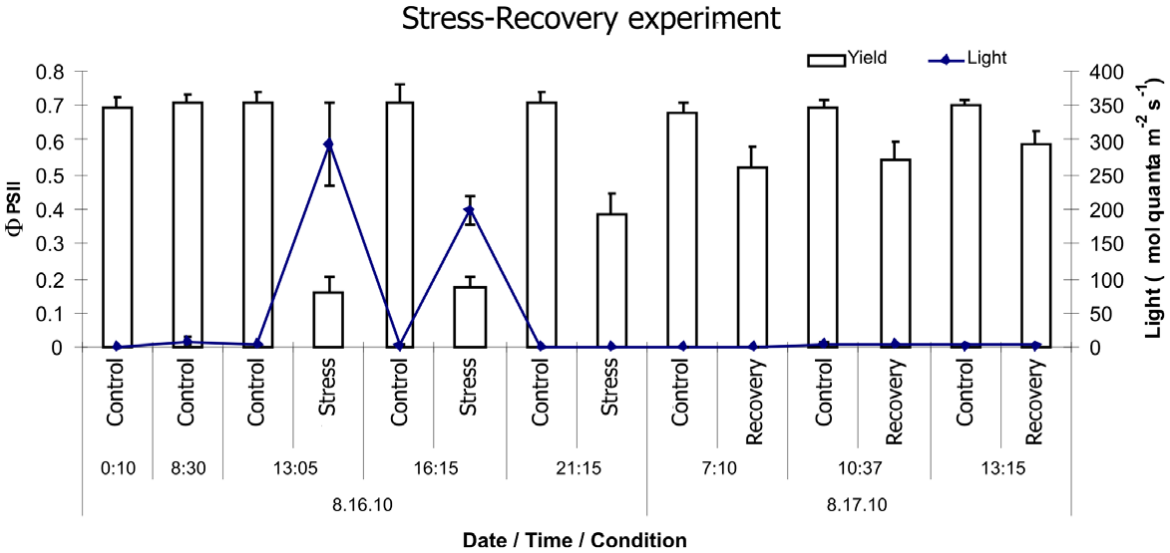
Chlorophyll *a* fluorescence, effective quantum yield (Φ_PSII_), of *in hospite Symbiodinium* during the Stress-Recovery experiment. Mean ± standard deviation of both effective quantum yield and light measurements taken for each fragment of *Porites astreoides* in the control, heat-light stressed and recovery treatments (n = 15) in flow-through systems supplied with sand-filtered sea water.

Stressed fragments were visibly bleached relative to their respective controls, and photosynthesis of their symbionts was still inhibited relative to their paired controls as the recovery period progressed (Fig. 3). Despite visible signs of bleaching, gene expression at the “recovery” time point was not significantly different between the control and heat-stressed fragments (Fig 2B–E “recovery”), with the exception of Hsp16, which was still up-regulated by ∼8-fold (P_MCMC_<0.01, Fig. 2A “recovery”). Significant treatment x time interactions for the heat-treated fragments between the “stress” and “recovery” time-points reflect this recovery of baseline gene expression patterns (Fig 2A–E).

### Double-gene assay for heat-light stress (Experiments 3 and 4)

**Figure 4:**
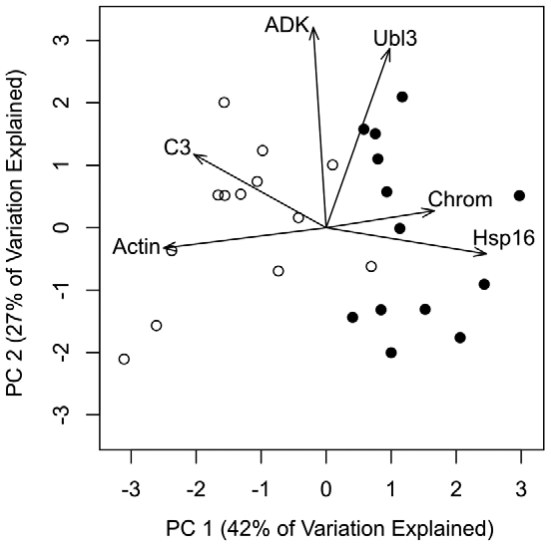
Principal components analysis of stress response gene expression in *Porites astreoides*. Black circles represent heat-light treated samples (n = 13), white circles represent control samples (n = 13). Vectors indicate loadings for each gene. The two major loadings on PC1 are Hsp16 (0.55) and actin (-0.56). Abbreviations are as follows: C3 =  complement component C3; ADK  =  adenosine kinase; Ubl3 =  Ubiquitin-like protein 3; Chrom  =  GFP-like chromoprotein.

After observing the remarkably consistent expression patterns obtained for Hsp16 and actin under heat-light stress during both years of our experiment, we decided to explore the potential to diagnose heat-light stressed colonies based on expression of a few key genes. In a principal components analysis of our first two experiments (1 and 2), 69% of the variance in our data was explained by the first two components, 42% by PC1 alone. Furthermore, heat-light treated fragments were fully distinguishable from controls (Fig 4). For PC1, we found Hsp16 to be the strongest positive loading and actin to be the strongest negative loading, at 0.55 and -0.56 respectively.

**Figure 5:**
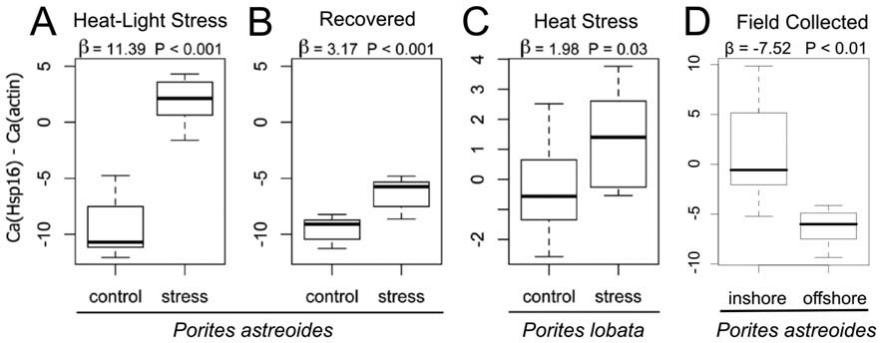
Application of double-gene assay to evaluate stress in *Porites astreoides* and *P. lobata*. In all panels, the vertical axis gives the difference between Ca values of Hsp16 and actin for (A) heat-light stressed and control *P. astreoides* (n = 15 pairs); (B) recovered and control *P. astreoides* (n = 15 pairs); (C) heat stressed and control *P. lobata* (n = 5 pairs) and (D) inshore (n = 9) and offshore (n = 7) *P. astreoides*. The text above the plots denotes the magnitude of change (β) according to the linear mixed model, and the significance of treatment (P). A thick black line indicates the median of the Ca difference values. The box represents the inter-quartile range (IQR) between the upper and lower quartile. The whiskers maximally extend 1.5 times beyond the IQR.

Since the expression changes of the two most responsive genes (actin and Hsp16) were inversely correlated, we designed a diagnostic assay based on the magnitude of difference between their expression levels. The difference between the non-normalized Ca's of actin and Hsp16, which we designated the “*Porites* Stress Index” (PSI), clearly distinguished between stress and control samples in our lab-based experiments with *P. astreoides* (Fig. 5A,B).

We also demonstrate successful transfer of this assay across species. Stressed corals were differentiated from controls in a lab-based experiment performed using an Indo-Pacific *Porites* species, *P. lobata*. The actin-Hsp16 Ca difference changed significantly (Fig. 5C), although not as dramatically as in experiments with *P. astreoides*. This reduced response is likely due to the fact that the control fragments were also stressed as all fragments were visibly paler at the end of experiment compared to the time of collection (data not shown).

Finally, we show that the PSI is able to discriminate between field populations experiencing different temperatures (Fig. 5D). At the time of field collections, water temperature of the inshore site was 32.2°C, while the offshore site was 31.5°C and mean temperatures for the three days surrounding sample collection were 32.1°C (±0.56°C) at the inshore site and 30.9°C (±0.44°C) at the offshore site. Consistent with this temperature difference, colonies of *P. astreoides* from the inshore site demonstrated a significantly higher PSI than those from the offshore site (P = 0.002, Fig. 5D). It is interesting to note that ambient light level at the turbid inshore site was less than at the offshore site, suggesting that temperature was the primary driver of the observed difference, if PSI is actually reflective of stress.

## Discussion

### Candidate genes and expression patterns

Over the past decade, sequence information has become available for over a half-dozen coral species comprising three of the most common reef-building genera (http://sequoia.ucmerced.edu/SymBioSys/index.php, http://www.bio.utexas.edu/research/matz_lab/matzlab/Data.html). Furthermore, a substantial body of literature has identified common patterns of gene expression regulation under stress conditions ranging from environmental pollutants [7], [8], [9], [10], [11] to thermal stress [12], [13], [14], [15]. Our results corroborate these findings and add a new super-responsive gene to the heat-shock panel, Hsp16. Previous studies have relied on larger heat shock proteins, such as Hsp60, 70 and 90, that display levels of up-regulation under heat stress on the order of 1 to 10-fold [12], [13], [14], [23]. We report similar levels of up-regulation for these large Hsps. However, when evaluating the expression of Hsp16, we observe a reproducible pattern of ∼800-fold up-regulation in heat-light stressed *P. astreoides* fragments relative to their paired controls.

Hsp16 is a cytosolic chaperone [38] that is highly sensitive to temperature-induced structural changes and is known to be involved in preventing protein aggregation [39]. In *Drosophila*, it has been shown that small to moderate increases in Hsp70 increase the thermotolerance response, while large increases reduce it, meaning that too much Hsp70 is detrimental to overall thermotolerance [40]. Therefore, the lack of extreme up-regulation in the large Hsps actually renders them rather poor markers of thermal stress events, despite being effective heat shock proteins. Hsp16 may be less damaging in high doses, or only induced during stress, which would explain the extreme up-regulation and subsequently rapid down-regulation when the event is over. To further evaluate its promising potential as a biomarker, we plan to explore whether the level of up-regulation of Hsp16 is proportional to the intensity of stress event, as well as the type of stressors that induce it.

Down-regulation of actin was our second most reproducible pattern over the two replicated Experiments (1) and (2). Consistent with DeSalvo *et al*. [12], we hypothesize that this expression pattern may be attributable to cytoskeletal changes in response to heat-light stress. Actin is an important cytoskeletal component involved in cell motility, growth and division [41]. Both microtubules and actin microfilaments have been shown to de-polymerize in response to heat shock in *Arabidopsis thaliana* [42], consistent with this hypothesis of cytoskeletal disruption in response to thermal stress. Conversely, the expression of a major cytoskeletal protein, such as actin, may be a proxy of the growth rate, in which case actin down-regulation could be indicative of overall growth inhibition in response to stress. This hypothesis can be tested in the future by correlating the actin expression in corals with DNA/RNA ratio, an accepted proxy of growth in other marine organisms [43], as well as directly by concurrent measurements of actin expression and growth in the lab under various conditions. While our data are consistent with the hypothesis of cytoskeletal re-organization in response to thermal/light stress, much work is still needed to understand the nature and the implications of this complex process.

Complement component C3 is an important factor in the innate immune response, whereby foreign cells are recognized, engulfed and destroyed [44]. We found significant down-regulation of C3 in response to heat-light stress in Experiment (1), and a trend towards down-regulation in the stress time-point of Experiment (2). The down-regulation of immunity as a result of thermal stress is an expected trade-off due to re-allocation of resources to counter immediate stress effects [45]. Rodriguez-Lanetty *et al*. [14] reported down-regulation of another putative immunity-related gene, a mannose-binding lectin, in *A. millepora* larvae exposed to thermal stress. The observation that coral disease events tend to follow thermal stress events, as has been reported in the Great Barrier Reef [46] and Caribbean, e.g. [47], may therefore be attributable to this down-regulation of immunity [14], [48], although some recent studies argue against such a scenario [49], [50].

We also observed significant up-regulation of a GFP-like chromoprotein as a result of heat-light stress in Experiment (1). Differential regulation of chromoproteins under heat stress is an increasingly common finding, though there are more reports of down-regulation [12], [16], [17], [18] than up-regulation [22]. Conversely, high light and ultraviolet radiation have been shown to result in up-regulation of GFP-like proteins in reef-building corals [51], [52]. Observation of up-regulation under heat-light stress could be due to the fact that GFP-like proteins have been hypothesized to participate in an oxidative stress response based on the superoxide-quenching properties of the jellyfish-derived GFP [53]. Also, Palmer *et al*. [54] have argued that GFP-like proteins may contribute to oxidative stress response through hydrogen peroxide scavenging. An alternative explanation of up-regulation of the GFP-like proteins could be their suggested role in photoprotection [55], [56] or otherwise modulating [57], [58] the photosynthesis of algal symbionts. Though this potential role remains controversial for a variety of reasons [59], [60], [61], the sheer prominence of GFP-like proteins in terms of expression level [62], [63] along with their clear propensity to respond to changing environmental conditions suggest that they may be important ecological indicators, and call for further investigation into their biological function.

### Recovery from heat-light stress

While extensive work has gone into characterizing gene expression patterns of scleractinians during immediate thermal stress events, to our knowledge, only one other study [13] has presented data on modulation of heat-light stress gene expression during a recovery phase in adult *Montastraea faveolata*. Contrary to our current results, DeSalvo *et al*. [13] reported minimal differences in gene expression patterns between stressed and recovered corals; though it is possible that the expression changes due to treatment were dampened because the magnitude of experimental stress was less than in the experiments reported here. In our stress-recovery experiment, for all genes showing differential regulation under immediate stress, the expression difference between stressed and recovered fragments was also significant in an apparently rapid recovery of homeostasis. For those fragments experiencing extreme thermal stress only twenty-four hours earlier, gene expression patterns between control and heat-light treated fragments were almost indistinguishable,, with moderate up-regulation of Hsp16 remaining as the only signature of the prior exposure. Comprehensive gene expression studies in yeast, which are also unable to thermo-regulate, have revealed that expression changes in response to many types of environmental stress are large, proportional to the intensity of the stress experienced and are transient [64]. Our expression data clearly recapitulate this pattern. Taken together with the Experiment (1) stress expression data, this suggests that our gene panel reflects acute rather than chronic stress, as the full magnitude of Hsp16 and actin regulation were observed on the first day of exposure during the stress-recovery experiment.

The rapid recovery of the “normal” gene expression levels observed in our experiment is also remarkable considering the fact that the recovering fragments were visibly bleached and the photosynthetic ability of their algal symbionts was still inhibited (Fig. 3). This indicates a possible discrepancy between the physiological states of the coral host and its symbionts, and suggests that the measures of coral stress relying only on symbiont parameters (such as degree of bleaching and photosynthetic efficiency) may not be fully informative for assessment of the holobiont's potential to survive stress.

### Double gene assay

The difference between expression levels of Hsp16 and actin (“*Porites* Stress Index”, PSI) emerged as a powerful indicator of acute heat-light stress, combining the dynamic ranges of the anti-correlated responses of these two genes (Fig. 5). The major advantage of such an assay is that there is no need for amplification and analysis of additional internal control (“housekeeping”) genes. Normalization is typically necessary because the measured abundance (Ca) of any gene is affected by template loading discrepancies between samples [65] in addition to variation due to gene regulation. Since two genes amplified from the same sample share the same template loading factor, it cancels out when the difference between their Ca values is computed, leaving only the biologically relevant variation [66]. The difference between the Ca values of actin and Hsp16 is negative for the non-stressed corals and approaches 0 or even becomes positive for stressed corals (Fig. 5). While the efficacy of this method over a broad range and intensity of stressors is still unclear, we believe it might present a minimally invasive means of rapidly evaluating coral stress *in situ*. Importantly, the assay is applicable to at least two species of *Porites*, an important reef-builder with a global distribution, and shows a between-population difference that is consistent with the observed temperature difference in the field. Future field studies will investigate the connection between physical characteristics of the environment and PSI. From the applied perspective, arguably the most important challenge is to understand how PSI measurements translate into bleaching, disease, and general mortality risks, for *Porites* spp. and for other corals growing on the same reef. If such connections are established, PSI could become a unique tool, facilitating environmental assessment from the point of view of the keystone organism, the coral itself.

## Supporting Information

Figure S1 **Temperature profile (°C) of Experiment 1**. Blue line: shaded control system, red line: sun-exposed system. Colony fragments were placed into the treatment flow-through system on Day 1. The vertical line marks the time of sampling.
(TIF)

Figure S2 **Chlorophyll** ***a*** **fluorescence, effective quantum yield (Φ_PSII_), of** ***in hospite Symbiodinium*** **during acclimation in Experiment 2**. Mean ± standard deviation of both effective quantum yield and light measurements taken for each *Porites astreoides* (n = 15) in the control flow-through system.
(TIF)

Figure S3 **Temperature (°C) and light (Log_10_Lumens) profile of Experiment 2**. Stress samples were taken at 14∶30 on 8/16. Recovery samples were taken at 14∶45 on 8/17.
(TIF)

Figure S4 **Evaluation of RNA quality among different preservation methods.** Duplicate samples from a single colony of *Porites astreoides* were fixed in either 96% ethanol (E), RNAlater (R), or snap-frozen in liquid nitrogen (Ln) and stored at −20°C for five days. The various preservatives were also compared to RNA extracted from non-fixed tissue (F). RNA was run on a 1% Agarose gel at 160 V for 25 minutes and illuminated under UV light. An additional aliquot was also run on a Bioanalyzer (Agilent) and the resulting RNA integrity (RIN) values are reported for each sample.
(TIF)

Figure S5 **Lonza Gel of RNA from samples used in experiment 2 (Stress-Recovery).** RNA (orange bands) is from samples at the stress time point. DNA appears as yellow bands. It is important to note that 3 µl of sample was loaded, regardless of concentration, therefore some samples appear brighter due to higher RNA amount. Rows with the same number indicate two fragments from the same colony exposed to either heat (H) or control (C) conditions. Empty wells and 2-log ladder are indicated by (B) and (L), respectively. A star above a sample indicates sufficient rRNA band quality for use in downstream reactions.
(TIF)

Figure S6 **Standard Q-Q plots of residuals from gene-wise linear mixed models on Experiment 2 data.** Quantiles of the residuals from our most sample-rich experiment (Experiment 2, “stress-recovery”) were plotted against the theoretical quantiles of the normal distribution. The gene names are indicated above each plot.
(TIF)
