# Supplementary figures and images for: Development of Gene Expression Markers of Acute Heat-Light Stress in Reef-Building Corals of the Genus Porites
Source: PLoS One. 2011 Oct 26;6(10):e26914. doi: 10.1371/journal.pone.0026914 (PMC3202587; doi:10.1371/journal.pone.0026914)

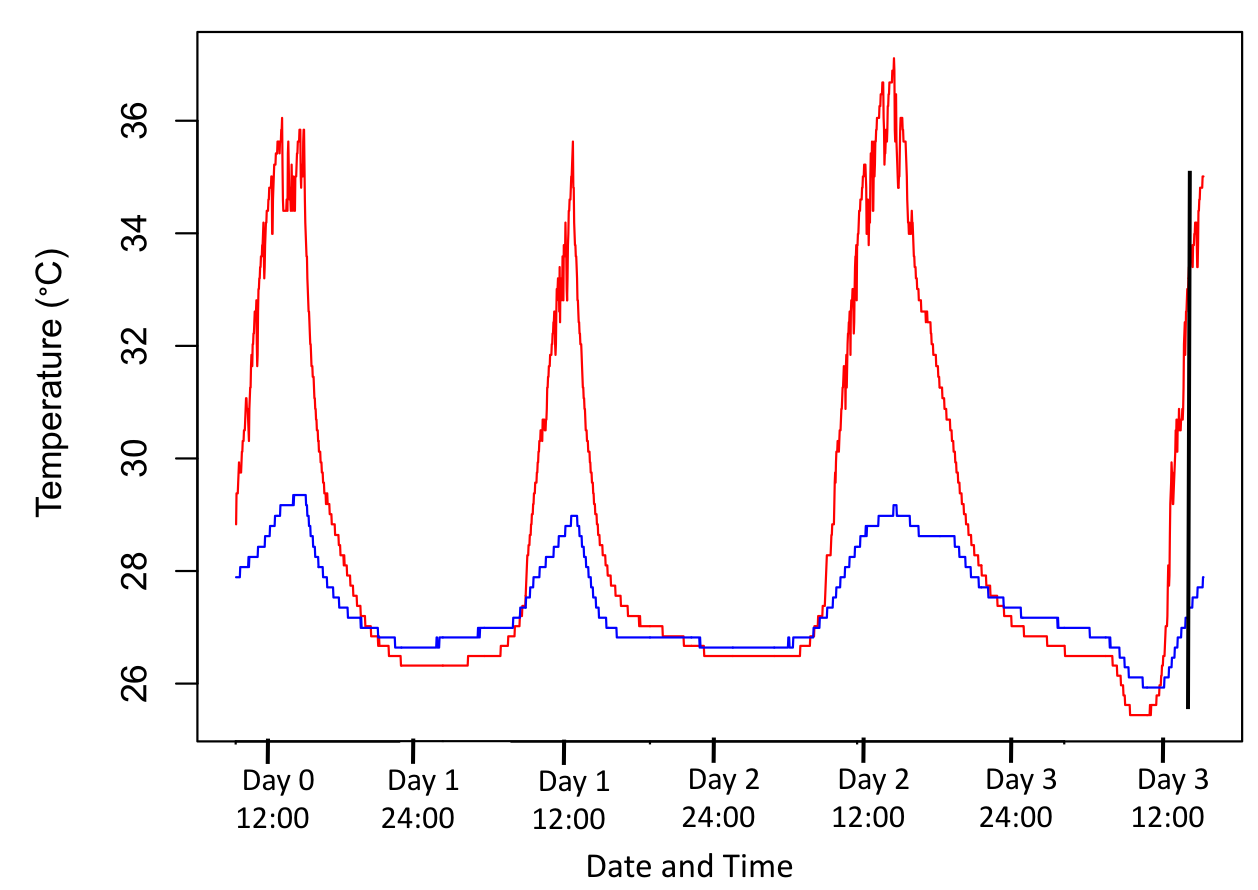

Supplement: Figure S1 — Temperature profile (°C) of Experiment 1. Blue line: shaded control system, red line: sun-exposed system. Colony fragments were placed into the treatment flow-through system on Day 1. The vertical line marks the time of sampling. (TIF) [file pone.0026914.s001.tif]

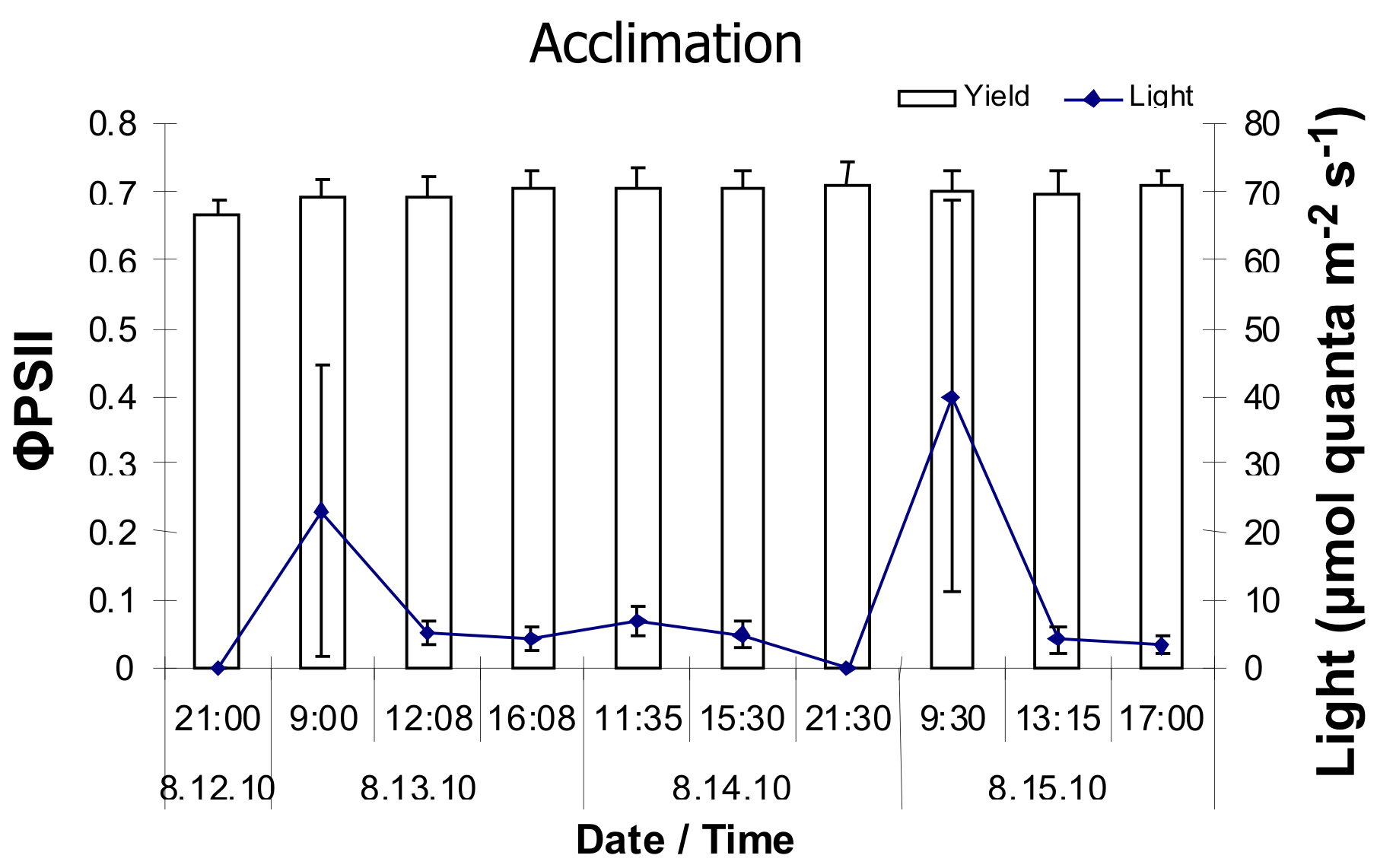

Supplement: Figure S2 — Chlorophyll a fluorescence, effective quantum yield (ΦPSII), of in hospite Symbiodinium during acclimation in Experiment 2. Mean ± standard deviation of both effective quantum yield and light measurements taken for each Porites astreoides (n = 15) in the control flow-through system. (TIF) [file pone.0026914.s002.tif]

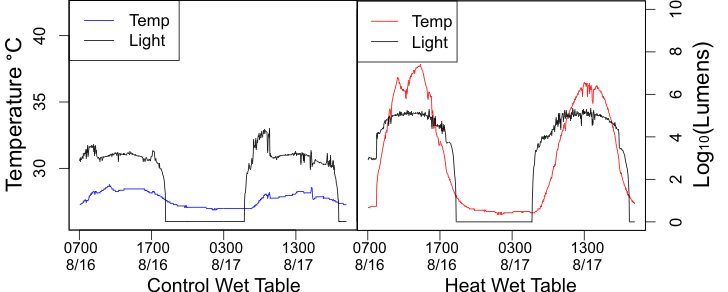

Supplement: Figure S3 — Temperature (°C) and light (Log10Lumens) profile of Experiment 2. Stress samples were taken at 14∶30 on 8/16. Recovery samples were taken at 14∶45 on 8/17. (TIF) [file pone.0026914.s003.tif]

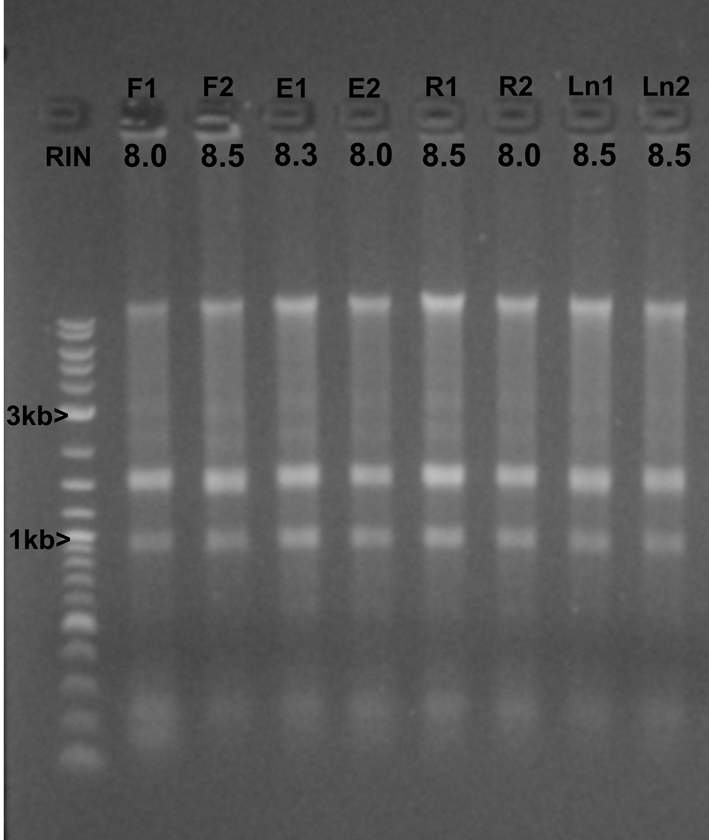

Supplement: Figure S4 — Evaluation of RNA quality among different preservation methods. Duplicate samples from a single colony of Porites astreoides were fixed in either 96% ethanol (E), RNAlater (R), or snap-frozen in liquid nitrogen (Ln) and stored at −20°C for five days. The various preservatives were also compared to RNA extracted from non-fixed tissue (F). RNA was run on a 1% Agarose gel at 160 V for 25 minutes and illuminated under UV light. An additional aliquot was also run on a Bioanalyzer (Agilent) and the resulting RNA integrity (RIN) values are reported for each sample. (TIF) [file pone.0026914.s004.tif]

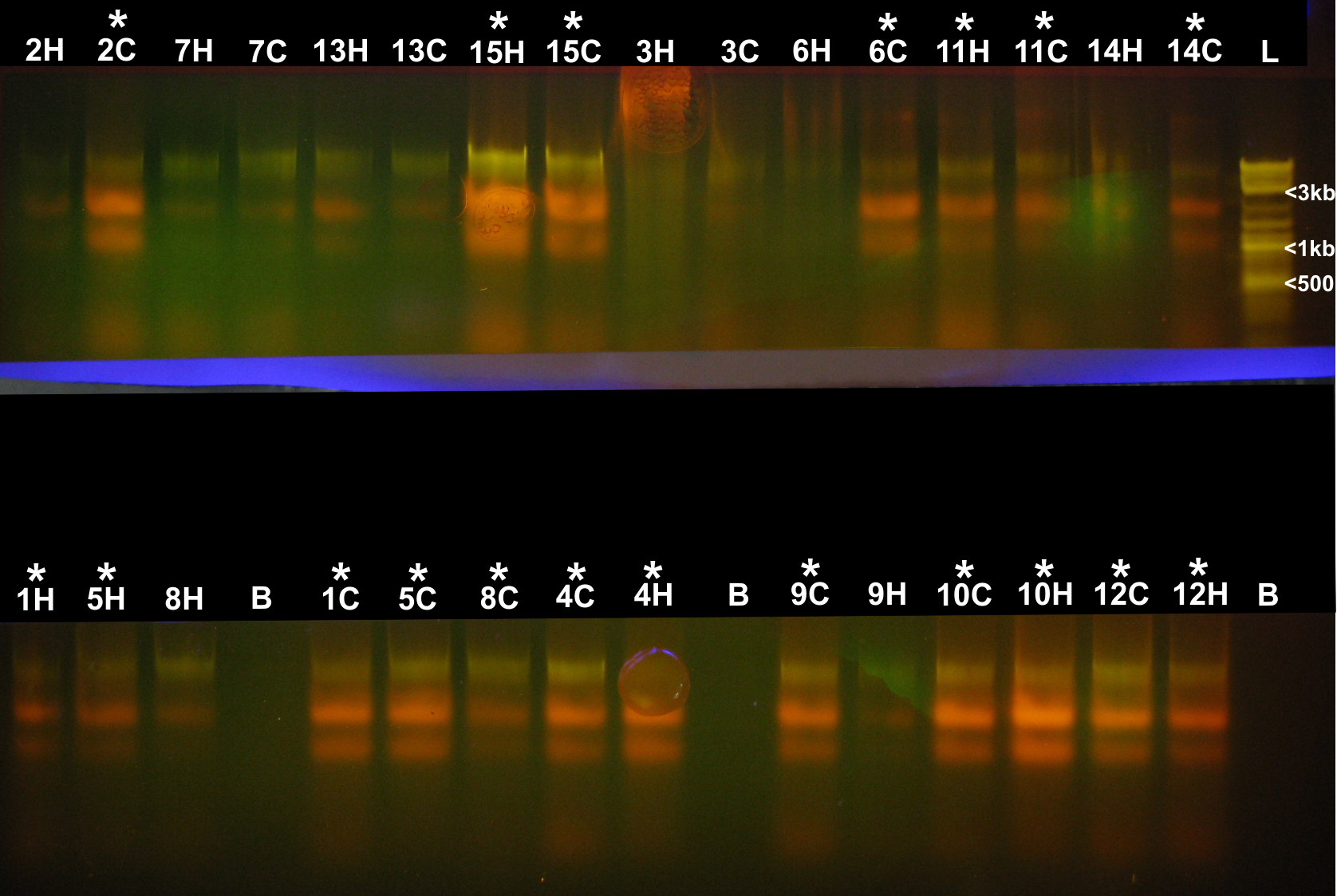

Supplement: Figure S5 — Lonza Gel of RNA from samples used in experiment 2 (Stress-Recovery). RNA (orange bands) is from samples at the stress time point. DNA appears as yellow bands. It is important to note that 3 µl of sample was loaded, regardless of concentration, therefore some samples appear brighter due to higher RNA amount. Rows with the same number indicate two fragments from the same colony exposed to either heat (H) or control (C) conditions. Empty wells and 2-log ladder are indicated by (B) and (L), respectively. A star above a sample indicates sufficient rRNA band quality for use in downstream reactions. (TIF) [file pone.0026914.s005.tif]

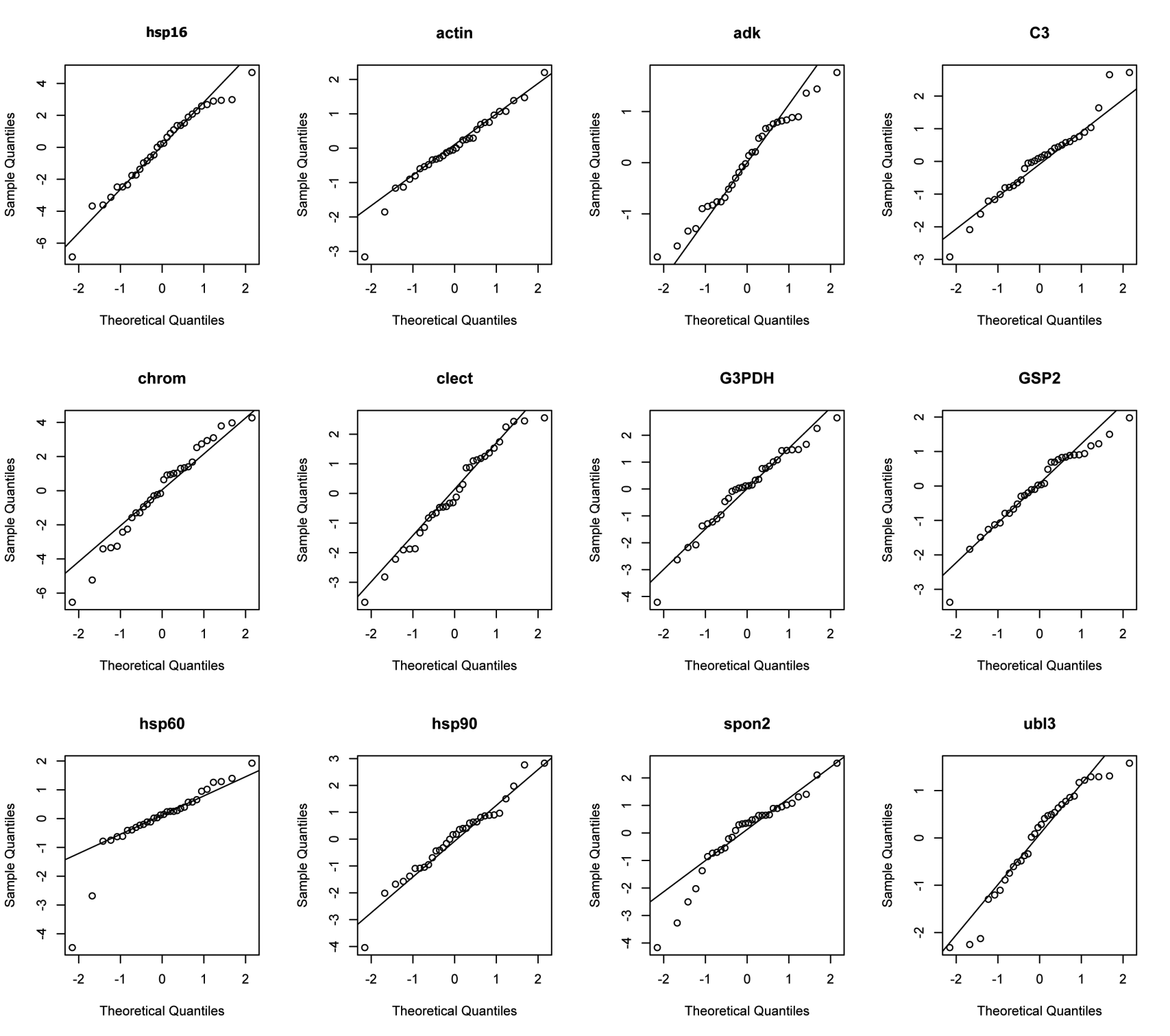

Supplement: Figure S6 — Standard Q-Q plots of residuals from gene-wise linear mixed models on Experiment 2 data. Quantiles of the residuals from our most sample-rich experiment (Experiment 2, “stress-recovery”) were plotted against the theoretical quantiles of the normal distribution. The gene names are indicated above each plot. (TIF) [file pone.0026914.s006.tif]
